# The effect of *Phoenix dactylifera* pollen on the expression of *NRF2, SOD2, CAT*, and *GPX4* genes, and sperm parameters of fertile and infertile men: A controlled clinical trial

**DOI:** 10.18502/ijrm.v19i6.9376

**Published:** 2021-07-27

**Authors:** Soghra Fallahi, Minoo Rajaei, Mohammad Javad Hesam, Mohsen Koolivand, Kianoosh Malekzadeh

**Affiliations:** ^1^Fertility and Infertility Research Center, Hormozgan University of Medical Sciences, Bandar Abbas, Iran.; ^2^International Institute of French Studies, University of Strasbourg, 2 Allée René Capitant, 67081, Strasbourg, France.; ^3^Department of Biochemistry, Faculty of Medicine, Hormozgan University of Medical Sciences, Bandar Abbas, Hormozgan, Iran.; ^4^Molecular Medicine Research Center, Hormozgan Health Institute, Hormozgan University of Medical Sciences, Bandar Abbas, Iran.; ^5^Department of Medical Genetics, Faculty of Medicine, Hormozgan University of Medical Sciences, Bandar Abbas, Iran.

**Keywords:** DPP, Male infertility, SOD2, NRF2, GPX4, CAT, ROS, Spermatozoa.

## Abstract

**Background:**

Oxidative stress is caused by the imbalance occurring between the creation and clearance of the reactive oxygen species (ROS), which is responsible for 30–40% of male infertility. The positive impact of *phoenix dactylifera* pollen (Date palm pollen, DPP) on the improvement of sperm parameters has been well documented in animal models.

**Objective:**

For evaluating the effect(s) of DPP on sperm parameters, ROS levels, expression of antioxidant genes, and activity of antioxidant enzymes of infertile men.

**Materials and Methods:**

In this controlled clinical trial, a total of 60 male case with infertility and 20 normospermic fertile men were recruited. Before and after the treatment with DPP, the case were administered 400 mg/kg of gelatinous capsules daily for 30 consecutive days and semen samples were taken. Quantitative real-time polymerase chain reaction was applied for the evaluation of the mRNA expression levels of Nuclear factor erythroid 2-related factor 2(*NRF2*), superoxide dismutase (*SOD2*), glutathione peroxidase 4(*GPX4*), and catalase (*CAT*) genes.

**Results:**

The *mRNA* expression levels of *NRF2, SOD2, GPX4*, and *CAT* (p < 0.05 for all) and significantly increased after treatment with DPP. The increased expressions of all antioxidant genes and enzymes significantly correlated with improvement in semen parameters including count (p = 0.01), motility (p = 0.05), and morphology (p = 0.01) of sperm. A significant correlation between the alteration of *SOD2* gene expression and SOD activity, *GPX4* and GPX, *and CAT* were also observed (p = 0.05).

**Conclusion:**

DPP can increase the expressions of *NRF2, GPX4, SOD2*, and *CAT* genes and also improve the semen quality in infertile men.

## 1. Introduction

As stated by the World Health Organization (WHO) guidelines, male infertility factors are responsible in roughly half of these couples (1). Moreover, male infertility has been proposed as one of the multifactorial disorders due to environmental, genetic, non-genetic factors, or a combination of these (2, 3). Several factors including structural reproductive tract abnormalities, mutations in mitochondrial DNA, chromosomal disorders, endocrine disturbances, hypogonadism, erectile dysfunction, chronic illnesses, several medications, exposure to radiation, reproductive tract infections, and cryptorchidism have previously been proposed as causes of male infertility (4, 5). However, the male infertility is still unknown in approximately 50% of the reported cases (4).

Accumulating evidence has shown oxidative stress (OS) as a major cause of the male infertility (6). OS has been reported to play an independent role as it has been considered as one of the conditions reflecting an imbalance between the production of oxygen-derived free radicals called ROS and the body's ability to the quenching of the ROS (7). In normal physiological conditions, ROS is generated at a low level in spermatozoa and plays a fundamental role in various biological mechanisms, namely capacitation, hyperactivation, and motility of sperms, acrosome reaction, and subsequent sperm–oocyte fusion and fertilization (8). Increased levels of ROS can pose a threat to sperms by causing peroxidation of sperm cellular membrane lipids, damage to sperm DNA integrity, decrease in sperm motility, oocyte–sperm fusion efficacy, and overall semen quality. Therefore, ROS plays a dual role as both deleterious and beneficial depending on their concentration in spermatozoa (9).

Moreover, enzymatic antioxidants like *SOD*, *CAT*, and *GPX*, which are abundant in the midpiece, make up a great proportion of human sperms (10). Nuclear factor erythroid 2-related factor 2 *(NRF2*), as a key protein in antioxidant defense system, acts as nuclear transcriptional factor and regulates the expressions of *GPX, SOD*, and *CAT* genes (11). Altered expressions of *NRF2, GPX4*, *SOD2*, and *CAT* genes can be associated with increased OS, which could contribute to impaired fertilization capacity and male infertility (12).

Experts have employed herbal remedies for treating various diseases especially male infertility since ancient times (13). Because of various antioxidants constituents, *Phoenix dactylifera* [Date Palm Pollen (DPP)], a good source of hormones, minerals, enzymes, vitamins, proteins, and fatty acids, has been used as an herbal remedy in the treatment of male infertility but very few data are available about its molecular nature (14). Besides, its efficacy has been studied in animal models (15), but few studies have addressed the effectiveness of DPP on humans. In a very recent study, DPP administration was proven effective on testosterone and follicle-stimulating hormone (FSH) levels, as well as sperm motility; however, the subjects of this study were sub-fertile (16). In addition, although some studies have evaluated the effect of DPP on sperm parameters (17), no previous study has addressed the influence of DPP on male fertility at genomic level.

Therefore, this research aimed at the determination of the impact(s) of DPP on the levels of *mRNA* expression of antioxidant genes (*NRF2, GPX4, SOD2*, and *CAT*) in infertile male.

## 2. Materials and Methods

### Study subjects 

**Figure 1:**
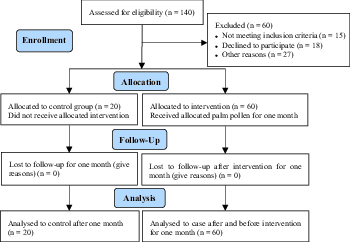
Consort flow chart.

This research randomized, single-blind, and comparative clinical trial included 60 case diagnosed with male infertility who were referred to the Om-e-Leila Fertility and Infertility Center, Om-e-Leila Hospital, Bandar Abbas, Iran, during 2016 (March)-17 (June) (Figure 1). We excluded the case with these criteria from the research: history of genetic and systemic disorders, reproductive tract abnormality, testicular trauma, alcohol and substance abuse, and fertility medications at least for six months before participation. DPP powder 400 mg/kg in gelatinous capsules was used to treat the eligible subjects daily for 30 consecutive days and all of the case finished the study course. DPP powder was purchased from palm farmers in autumn and formed into capsules in the laboratory. Additionally, 20 fertile males who had fathered a child within the last 2 yr and their semen samples were recruited as the controls. Initially, the controls comprised of the same number of case as the case group, however, due to the inconvenience of sample collection for semen analysis, some case failed to cooperate and were excluded from the study. At the time of enrollment, demographic data were obtained from all case using a structured interview form.

### Sample collection and isolation of spermatozoa from seminal fluid

Semen samples were obtained twice from cases, before and after the treatment period 30 consecutive days, and once from controls. Then, we collected the fresh semen samples in the sterile plastic containers by masturbation after three to five days of sexual abstinence. Then, we liquefied the samples at 37°C for 30 min and semen analysis (semen volume, pH, viscosity, sperm morphology, concentration, and motility) was immediately performed with the use of the Sperm Quality Analyzer IIC (SQA IIC, United Medical Systems Inc., Santa Ana, CA, USA) based on the WHO guidelines (18). In addition, in this randomized, single-blind, and comparative clinical trial, Semen analysis was performed by the blind technician. After purification of spermatozoa by Goodrich methods (19), BSA-free Ham's-F10 medium was used to wash the samples twice and then stored in RNALater solution (Qiagen: Germany) at a temperature of –80°C until RNA extraction. Sperms with smooth, rimmed, oval-shaped heads, 2.5–3.5 µm wide and 5–6 µm long, free of large vacuoles, an acrosome that covers 40–70% of the head, a midpiece of nearly the same length as the head but much slimmer, and un-coiled 45 µm-long tail thinner than the head and midpiece, and with no defects in the tail or head under light microscope are considered as having normal morphology.

### Purification and measurement of free 8-Isoprostane

An affinity column (Cayman Chemical, Ann Arbor, MI, USA) available in the market was used to purify the free 8-Isoprostane in duplicate. For isolating of precipitates, all specimens were first centrifuged at 15000 g. Then, we used column buffer to dilute supernatant 1:5 and performed the next procedures based on the manufacturer's protocol. Concentration of 8-Isoprostane was assessed using 50 ml from specimens at a wave length of 405 nm by the enzyme-linked immuno-sorbent assay (ELISA) reader (STAT FAX 2100, USA).

### RNA extraction and cDNA synthesis

NucleoSpinⓇ RNA Midi kit (Macherey-Nagel, Düren, Germany) was used to extract the total RNA from the samples and then RNase-free DNase I (Thermo Scientific, USA), based on the manufacturer's manual, was used to treat the RNA yield. After that, agarose gel electrophoresis was employed for confirming the quality of the extracted RNA. Its quantity was confirmed using Nano DropⓇ ND-1000 Spectrophotometer (Thermo Scientific, USA). In accordance with the company's guidelines, we applied the Revert Aid${}^{\mathrm{TM}}$ First Strand cDNA Synthesis Kit (Fermentas, Canada) to reverse transcribe 20 ng of total RNA to cDNA.

### Quantitative real-time polymerase chain reaction

Based on the company's instructions, we carried out duplicate real-time PCR with the gene-specific primers by means of SYBRⓇ Premix Ex Taq${}^{\mathrm{TM}}$ II (Tli RNaseH Plus) kit (Takara Bio Inc., Shiga, Japan) produced by the Rotor-GeneTM 6000 system (Corbett Research: Australia). Thermal cycling consisted of a round of denaturation for 30 sec at 95°C, which was followed by 40 more 5-sec cycles at 95°C. Each primer pair was heated at their specific temperatures (shown in Table I) for 15 sec and another 20-sec round at 72°C. Using serial dilution of cDNA, standard curves were drawn and thus level of the genes' expression level was normalized based on the mean expression of β-actin (the housekeeping gene). No template negative control was used.

**Table 1:** Primer sequences and real-time PCR condition


**Gene**	**The primers sequences (5' →3')**	**Amplicon (bp)**	**Annealing Temp. (°C)**
*SOD2*	F-TTGACAAGTTTAAGGAGAAGC R-CTGAAGGTAAGCGTGC	197	59
*GPX*	F-CCGATACGCTGAGTGTGGTT R-CTCCCTGGCTCCTGCTTCC	72	61
*CAT*	F-CTGGGACTTCTGGAGCCTAC R-TCTCGCCGCATCTTCAACA	211	59
*NRF2*	F-CCAGCACATCCAGTCAGAA R-CGTAGCCGAAGAAACCTCA	151	58
*β-actin*	F-GCCTTTGCCGATCCGC R-GCCGTAGCCGTTGTCG	90	59
SOD2: Superoxide dismutase 2, GPX: Glutathione peroxidase, CAT: Chloramphenicol acetyltransferase, NRF2: Nuclear factor erythroid 2-related factor 2			

### Ethical considerations

The Ethics and Human Rights Committee of Hormozgan University of Medical Sciences (under the ethics code: HEC-93-12-4) verified this research, and then the written informed consents were received from all participants after the purpose of study was explained based on the declaration of Helsinki.

### Statistical analysis

According to the research design, SPSS 16.0 (SPSS Inc., Chicago, IL: USA) was employed for statistical analyses and finally we set p < 0.05 to be statistically significant. Then, we employed a paired *t* test for comparing the sperms' parameters before and after the treatment period. In addition, we applied student's *t* test to compare the sperm parameters between controls and case (after treatment). Analysis of the distribution of *NRF2, GPX4, SOD2*, and *CAT* genes expression levels with the use of the Kolmogorov–Smirnov test indicated the lack of normal distribution of the data. For this reason, we employed non-parametric statistical tests for comparing the data. The Wilcoxon two-sample test was applied to detect the significant differences found in the *NRF2, GPX4, SOD2*, and *CAT* genes expression levels before and after the treatment. To compare the expression levels of the genes between the treated and healthy control individuals, two-tailed Mann–Whitney U-test was used. Spearman's Rho correlation test and was also utilized to analyze the correlations between the *NRF2, GPX4*, *SOD2, *and *CAT* genes expression levels and the various sperm parameters and free 8-Isoprostane levels.

## 3. Results

Demographic information of the participants are demonstrated in Table II and also change information the sperm count, semen volume and other parameters of case significantly enhanced after treatment with DPP. Comparison of the mean concentration of free 8-isoprostane and its significant reduction after capsule treatment was one of the most significant results of this study (p < 0.05) (Table III). Next, we assessed the possible correlation between the 8-Isoprostane concentration and seminal parameters. The common frequency of abnormal parameters of the participants in this study is also mentioned (Table IV).

**Figure 2:**
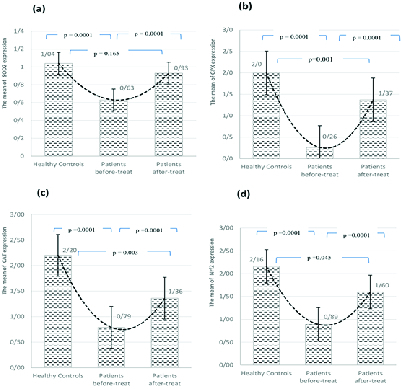
Changes in mRNA level of SOD2 (a), GPX (b), CAT (c), and NRF2 (d) genes before and after the treatment of infertile men with DPP in comparison with healthy controls and cases.

Comparative evaluation of mRNA expression of NRF2, GPX4, SOD2 and CAT genes before and after DPP treatment and in healthy individuals using quantitative real-time PCR and evaluation of the ratio of these genes at the mRNA level using the 2 - ΔΔct method significantly increased showed the expression of these genes. The mRNA expression levels of NRF2, GPX4, and CAT genes in healthy control was significantly higher than the case after the treatment (p = 0.04, p < 0.05, and p = 0.03) (Figure 2) However, the differences between the mRNA expression levels of SOD2 gene in healthy controls and case after treatment with DPP were not statistically significant (p = 0.16).

Examination of seminal enzymatic activity results of GPX4, SOD2 and CAT genes in in case and healthy controls showed that the mean activity of seminal GPX4, SOD2 and CAT enzymes were higher in controls compared with case, in addition Our findings showed that the mean activity of GPX4, SOD2, and CAT enzymes were higher in case after the treatment compared with case before the treatment (Table V).

The associations between fold changes of NRF2, GPX4, SOD2, and CAT genes expressions with sperm parameters in this article indicated that increase in the NRF2, GPX4, SOD2, and CAT genes expressions after treatment with DPP is significantly associated with increased the sperms' count, motility, and volume, and improved morphology of sperm (Table VI).

Spearman's correlation analysis showed SOD2 mRNA expression was significantly correlated with count, motility, and morphology of sperm, moreover, we found a relationship between the GPX4 expression at mRNA level and sperm count, sperm motility, morphology of sperm, pH of semen fluid, and seminal plasma free 8-Isoprostane level. The CAT expression at mRNA level was significantly related to the count of sperm, motility, and morphology of sperm and seminal plasma free 8-Isoprostane level (Table VII). We also detected a positive correlation between the NRF2 mRNA expression levels and sperm count, sperm motility, appearance and morphology of sperm, and pH of semen fluid.

**Table 2:** Demographic properties of case and healthy controls


**Variable**	Case			**Control**		
	**N (%)**	**Mean ± SD**	**Range**	**N (%)**	**Mean ± SD**	**Range**
**Age (yr)**						
**≤ 30**	40 (66.67)	27.62 ± 2.23	22.00–30.00	12 (60.00)	27.75 ± 2.00	24.00–30.00
**> 30**	20 (33.33)	34.35 ± 3.32	31.00–40.00	8 (40.00)	33.62 ± 1.92	32.00–37.00
**Weight (Kg)**	60 (100.00)	88.36 ± 1.49	60.00–110.00	20 (100.00)	85.95 ± 9.68	60.00–104.00
**Height (M)**	60 (100.00)	1.66 ± 0.08	1.40–1.85	20 (100.00)	1.70 ± 0.07	1.57–1.81
BMI*						
**Normal**	15 (15.00)	22.11 ± 2.19	18.52–24.86	6 (30.00)	22.26 ± 1.90	18.63–24.11
**Overweight**	35 (58.33)	27.13 ± 1.17	25.00–29.71	8 (40.00)	25.54 ± 1.82	22.81–29.21
**Obesity grade 1**	10 (16.67)	31.83 ± 1.52	30.92–34.48	4 (20.00)	33.01 ± 0.56	30.42–34.78
**Obesity grade 2**	–	–	–	2 (10.00)	39.21 ± 0.06	39.06–39.36
*BMI: Body mass index, Normal (<18 – ≤ 24.99), Overweight (≤ 25 – ≤ 29.99), Obesity grade 1 (≤ 30 – ≤ 34.99), Obesity grade 2 (≤ 35 – ≤ 39.99)						

**Table 3:** The comparison of sperm parameters and free 8-Isoprostane levels between the patients after treatment and healthy control


**Variable**	**Cases after treatment**	**Controls**	P-value*
**Count**	33.21 ± 25.94	77.15 ± 22.63	≤ 0.001
**Volume**	3.71 ± 1.32	2.65 ± 0.74	≤ 0.001
**Appearance**	–	–	0.99
**Viscosity**	–	–	0.56
**Liquefaction**	–	–	0.99
**pH**	7.58 ± 0.16	7.67 ± 0.27	0.26
**Motility**	34.06 ± 13.84	61.36 ± 11.89	≤ 0.001
**Morphology**	32.18 ± 9.98	61.90 ± 9.49	≤ 0.001
**Free 8-Isoprostane**	2.05 ± 2.01	1.90 ± 1.02	0.31
Data are expressed as the Mean ± standard deviation (SD), *Analyzed by Student's *t* test			

**Table 4:** The comparison of sperm parameters and free 8-Isoprostane levels of case before and after the treatment


**Variable**	**Before treatment**		**After treatment**		P-value*
	**N (%)**	**Mean ± SD**	**N (%)**	**Mean ± SD**	
**Count**					
**Normal ( ≥ 15 million/ml)**	15 (25.00)	16.33 ± 1.30	49 (81.67)	39.18 ± 25.03	< 0.001
**Abnormal ( < 15 million/ml)**	45 (75.00)	5.84 ± 4.10	11 (18.33)	6.62 ± 3.50	
**Volume**					
**Normal ( ≥ 1.5 ml)**	52 (86.67)	3.38 ± 1.33	60 (100.00)	3.71 ± 1.32	< 0.001
**Abnormal ( < 1.5 ml)**	8 (13.33)	1.17 ± 0.17	–	–	
**Appearance**					
**Normal**	56 (93.30)	–	60 (100.00)	–	0.12
**Abnormal**	4 (6.70)	–	–	–	
**Viscosity**					
**Normal**	59 (98.30)	–	60 (100.00)	–	0.99
**Abnormal**	1 (1.70)	–	–	–	
**Liquefaction**					
**Normal**	57 (95.00)	–	59 (98.30)	–	0.5
**Abnormal**	3 (5.00)	–	1 (1.70)	–	
**pH**					
**Normal**	59 (98.30)	7.70 ± 0.22	60 (100.00)	7.58 ± 0.16	≤ 0.001
**Abnormal**	1 (1.70)	–	–	–	
**Motility**					
**Normal (≥ 40%)**	15 (25.00)	46.13 ± 11.23	20 (33.33)	49.05 ± 10.96	< 0.001
**Abnormal (< 40%)**	45 (75.00)	22.68 ± 8.15	40 (66.67)	26.57 ± 7.66	
**Morphology**					
**Normal (≥ 4%)**	6 (10.00)	42.00 ± 3.94	15 (25.00)	45.40 ± 5.08	< 0.001
**Abnormal (< 4%)**	54 (90.00)	24.35 ± 7.82	45 (75.00)	27.77 ± 6.78	
**Free 8-Isoprostane (ng/ml)**	60 (100.00)	4.37 ± 4.13	60 (100.00)	2.05 ± 2.01	< 0.001
*Wilcoxon test					

**Table 5:** The mean of SOD, GPX, CAT, and NRF2 genes expression and the activity of their enzymes in case (before and after the treatment) and healthy controls


		Case			
**Genes**		**Before**	**After**	P-value*	**Healthy controls**	P-value**
		**The mRNA expression levels**						
	**SOD2**	0.63 (0.01–1.29)	0.93 (0.11–2.22)	< 0.001	1.04 (0.54–1.69)	0.16
	**GPX**	0.26 (0.01–1.06)	1.37 (0.48–3.70)	< 0.001	2.00 (1.09–4.00)	< 0.001
	**CAT**	0.79 (0.03–1.55)	1.36 (0.24–2.96)	< 0.001	2.20 (0.98–4.53)	≤ 0.001
	**NRF2**	0.89 (0.11–2.64)	1.60 (0.29–3.20)	< 0.001	2.16 (0.26–3.93)	0.04
**The enzyme activity**						
	**SOD2**	75.96 (6.77–181.31)	82.78 (32.60–190.1)	0.030	112.29 (75.35–341.33)	≤ 0.001
	**GPX**	53.22 (20.27–110.10)	73.70 (22.17–208.42)	< 0.001	107.39 (54.13–218.39)	< 0.001
	**CAT**	1.69 (0.01–5.98)	2.53 (0.29–7.83)	< 0.001	4.08 (1.89–6.43)	< 0.001
Data presented as mean (min–max), *Analyzed by Wilcoxon test, **Analyzed by Mann–Whitney test, SOD2: Superoxide dismutase 2, GPX: Glutathione peroxidase, CAT: Chloramphenicol acetyltransferase, NRF2: Nuclear factor erythroid 2-related factor 2						

**Table 6:** The mean of *SOD*, *GPX*, *CAT*, and *NRF2* genes fold change in relation to abnormal seminal fluid parameters before and after the treatment


	*SOD2*			*GPX4*			*CAT*			*NRF2*		
	**Before**	**After**	P-value*	**Before**	**After**	P-value*	**Before**	**After**	P-value*	**Before**	**After**	P-value*
**Count **	0.6371	0.9500	< 0.001	0.2840	1.3989	< 0.001	0.8089	1.3980	< 0.001	0.8867	1.6698	< 0.001
**Volume**	0.4925	0.8750	0.012	0.2450	1.7300	0.012	0.8963	1.5425	0.012	0.7900	1.8750	0.01
**Appearance**	0.6450	0.8800	0.066	0.2425	1.4900	0.068	0.6775	1.7175	0.068	0.4400	1.2650	0.06
**Liquefaction**	0.2633	0.6337	0.109	0.2237	1.3567	0.109	0.5467	1.2900	0.109	0.3233	1.0967	0.10
**Motility **	0.6904	0.9740	0.012	0.8633	1.3460	0.024	1.1209	1.3662	0.041	1.2360	1.6782	0.03
**Morphology **	0.6309	0.9624	< 0.001	0.2706	1.3828	< 0.001	0.7872	1.3881	< 0.001	0.8717	1.6439	< 0.001
${}^{*}$Analyzed by paired *t *test, SOD2: Superoxide dismutase 2, GPX: Glutathione peroxidase, CAT: Chloramphenicol acetyltransferase, NRF2: Nuclear factor erythroid 2-related factor 2												

**Table 7:** The correlation between *SOD2*, *GPX4*, *CAT*, and *NRF2* genes expression levels with sperm parameters


	*SOD2*		*GPX4*		*CAT*		*NRF2*	
	**rs**	**P-value**	**rs**	P-value*	**rs**	**P-value**	**rs**	P-value*
**Count **	0.339	0.0001${}^{*}$	0.610	0.0001${}^{*}$	0.410	0.0001${}^{*}$	0.477	≤ 0.001
**Volume**	0.016	0.853	0.036	0.675	0.029	0.733	0.149	0.07
**Appearance **	–0.076	0.373	–0.161	0.057	–0.148	0.081	–0.203	0.01${}^{*}$
**Liquefaction **	–0.153	0.073	–0.128	0.131	–0.162	0.071	–0.159	0.06
**Viscosity**	–0.142	0.094	–0.096	0.262	–0.093	0.272	–0.109	0.19
**PH**	–0.142	0.094	–0.194	0.022${}^{*}$	–0.088	0.300	–0.281	≤ 0.001
**Motility **	0.205	0.015${}^{*}$	0.422	0.001${}^{*}$	0.211	0.012${}^{*}$	0.282	≤ 0.001
**Morphology **	0.261	0.002${}^{*}$	0.485	0.0001${}^{*}$	0.273	0.001${}^{*}$	0.243	≤ 0.001
**8-Isoprostane**	–0.108	0.202	–0.259	0.002${}^{*}$	–0.194	0.021${}^{*}$	–0.119	0.16
${}^{*}$Analyzed by Spearman's Rho, SOD2: Superoxide dismutase 2, GPX: Glutathione peroxidase, CAT: Chloramphenicol acetyltransferase, NRF2: Nuclear factor erythroid 2-related factor 2, rs: Spearman's correlation coefficient								

## 4. Discussion

As mentioned earlier, excessive levels of ROS can negatively affect the process of spermatogenesis and impair the ability of spermatozoa to fertilize ova (9). Antioxidants and antioxidant enzymes in the male reproductive tracts are the main antioxidant defense systems that protect spermatozoa from oxidative damage caused by ROS (6, 9). Therefore, antioxidant therapy could be beneficial in increasing the scavenging capacity of seminal plasma and improving the semen quality in infertile males. In traditional medicine, DPP has an extensive utilization as one of the folk remedies to treat the male infertility (14). This study was designed for investigating the *mRNA* expression levels of the antioxidant genes *NRF2, GPX4, SOD2*, and *CAT* in infertile males before and after treatment with DPP and healthy controls. We also investigated the impact of DPP on the sperm parameters of infertile man. We found that taking DPP in infertile males significantly improves the semen quality including sperm count, semen volume, morphology, and motility of sperm (p < 0.05) (Table IV). In accordance with our data, Al-Snafi reported that the sperms' motility and count enhanced in the infertile males who were given treatment with DPP (20). Similarly, evaluated the effects of DPP on the sperm parameters of infertile man; sperm motility, count, morphology, as well as forward-progressive motility significantly increased with DDP (21). Al-Dujaily also reported a significant improvement in the sperms' motility and count after the addition of DPP extract to sperm activation medium (22). In a study on adult male Sprague–Dawley rats, it was found that DPP can promote the count, motility, morphology, and DNA quality of sperms. Moreover, it increased the weight of testis and epididymis (23). Adaay and Mattar showed that after treatment with the extracts, a mixture of 3 plants (*P. dactylifera*, *Nasturtium officinale* and *Tribulus terrestris*), the sperm concentration and motility increased remarkably in Swiss albino male (24). These findings suggest that consumption of DPP in infertile males of human and animal models improves a number of semen parameters through various possible molecular mechanisms. Analysis of seminal plasma has revealed that human semen contains high concentrations of mineral ions such as calcium (Ca) and zinc (Zn) (24), which fundamentally contribute to spermatogenesis. In sperm cells, a major component of the signaling cascade leading to capacitation, hyperactivation, chemotaxis, and acrosome reaction is Ca. There exists convincing data supporting the critical role of the Zn in mediating of nuclear chromatin decondensation, sperm oxygen consumption, and acrosin activity (25). Additionally, it has been documented that the zinc finger proteins are involved in the expression of steroid hormone receptors (26). Selenium (Se), as essential micronutrients, acts as free radical scavenger and improves semen quality to the presence in antioxidant component of selenoprotein (27). DPP contains many minerals including Ca, Zn, and Se; therefore, it can positively influence the quality of semen. However, other possible mechanisms of sperm quality improvement by DPP need to be further looked into. Moreover, this might be the reason for similar findings in previous studies of animal models which were in line with our results.

According to the findings, *mRNA* expression levels of antioxidant genes *SOD2, GPX4, CAT* and *NRF2 *are significantly lower in infertile case in comparison with the healthy control. Moreover, *mRNA* expression levels of *NRF2, GPX4, SOD2, *and *CAT* genes increased in case after treatment with DPP (p < 0.05) (Figure 2; Table III). Similarly, reported a link between functional polymorphisms in *NRF2* promoters and dysfunctional spermatogenesis in humans. Due to the role of *NRF2* in the transcription of some antioxidant genes such as *GSTM1 *and *SOD2 mRNA*, functional polymorphisms in *NRF2* promoters can decrease their levels (13). Previous findings have shown, that decreased fertility in male *NRF2* (–/–) compared to the wild-type is a result of knockout of *NRF2* transcription factor and the subsequent disruption in spermatogenesis (28). In light of these studies and our findings, sperm fertilization capacity is determined by the level of *NRF2* expression. The expression of *GPX4* appears to increase in testis during spermatogenesis (28). *GPX4* is predominantly localized in the mitochondria of spermatozoa and through the antioxidant activity against mitochondrial ROS and by maintaining the integrity of the structure of mitochondrial capsule, it plays an important role in sperm motility (29). Diaconu and coauthors reported a substantial decrease in expressing *GPX4* in spermatozoa of the human infertile males who have been diagnosed with oligoasthenozoospermia (30). Imai et al. reported an association between severe abnormalities in spermatozoa and depletion of *GPX4* in spermatocytes (29). Furthermore, it has been shown that *GPX4* is involved in condensation of chromatin and protection of sperm DNA against oxidation, a necessary process in the maintenance of sperm quality (30). It has been reported that mitochondrial *GPX4* disruption causes abnormalities in spermatozoa (31). SOD2, mitochondrial SOD or Mn SOD, is one of the highly expressed genes that are regulated by the *NRF2-ARE* signaling pathway in human semen that are responsible for protecting the sperms from oxidative damages (32). Some have also shown that SOD contribute physiologically to the maintenance of a balance between H${}_{2}$O${}_{2}$ and O${}^{2-}$ and disruption in the expression of the enzyme relate to the impaired functions of the sperms (33). Another study reported the involvement of the decreased seminal plasma scavenger antioxidant capacity, especially as the lower SOD activity, in the male infertility (34). In fact, the existence of nearly 1 Ala-MnSOD allele (rs4880) enhanced the risks of infertility in male subjects (35). Other authors demonstrated the positive association between the seminal SOD activity and the sperm fertilization potential and male infertility (36). It has been showed that the presence of variant Val allele (Ala16Val polymorphism in the SOD2 gene) can alter the conformation of the secondary structure of the protein leading to decreased efficiency of transport into mitochondria which is believed to be associated with infertility (37). Moreover, a positive correlation between the protein expression of *CAT* and sperm motility and morphology was observed in a study performed by Macanovic and colleagues (38). It has been reported that functional polymorphism in *CAT* gene (–262 C/T) alters the level of *CAT* in blood and influences the promoter activity (39). Sabouhi and colleagues demonstrated that this mutation is correlated to the males' infertility (40). Taken together, results reveal the fundamental contribution of expression and activity of antioxidants enzymes to the pathogenesis of male infertility. Results of a systematic review revealed that DPP can be an appropriate supplement for the treatment of infertility, in that it can decreases free radicals, increases sperm motility, and enriches sperms with important minerals (17). All in all, consistent with the previous studies, our results demonstrate a significant improvement of sperm parameters and antioxidant gene expression by DPP in case who suffer from male infertility.

It has been demonstrated that DPP contains notable amounts of polyphenols including phenolic acids, flavonoid glycosides, and anthocyanidins (41). Yeh and colleague reported that gallic acid (a phenolic acid) induces an accumulation of *NRF2* (42). Vari and colleague revealed that protocatechuic (another phenolic acid) can improve the antioxidant potential of the macrophage through a pathway in which *JNK-mediated NRF2* activation plays a critical role (43). Because of the regulation of expression of several antioxidant enzymes such as *GPX4*, *SOD2*, and *CAT by NRF2*, it is not surprising that the elevated expression of *NRF2* lead to increase in the expressions of antioxidant genes *GPX4, SOD2, *and *CAT*. One limitation of the current study was that the treatment was given to the infertile subjects for a duration of one month due to their lack of cooperation and limited resources for the study; however, the minimum time required for a complete cycle of spermatogenesis is 86 days. Therefore, future studies should consider a three-month treatment period for better results.

## 5. Conclusion

We could present further documents for supporting this hypothesis: altered expression of antioxidant genes *NRF2, GPX4, SOD2, *and *CAT* at *mRNA* levels are correlated to the greater risks of males' infertility. We also conclude that administering DPP in infertile males enhances the levels of expression of *NRF2, GPX4, SOD2, *and *CAT* genes and improves the semen quality including sperm count, semen volume, and morphology and motility of sperm.

##  Conflict of Interest

Hereby, it is declared that there is no conflict of interest regarding the research.

## References

[B1] Agarwal A, Mulgund A, Hamada A, Chyatte MR (2015). A unique view on male infertility around the globe. Reprod Biol Endocrinol.

[B2] Poongothai J, Gopenath TS, Manonayaki S (2009). Genetics of human male infertility. Singapore Med J.

[B3] Shamsi MB, Kumar K, Dada R (2011). Genetic and epigenetic factors: Role in male infertility. Indian J Urol.

[B4] Kumar N, Singh AK (2015). Trends of male factor infertility, an important cause of infertility: A review of literature. J Hum Reprod Sci.

[B5] SkakkebÃ¦k NE, JÃ¸rgensen N, Main KM, Meyts ERD, Leffers H, Andersson AM, et al

[B6] Agarwal A, Virk G, Ong C, Du Plessis SS (2014). Effect of oxidative stress on male reproduction. World J Mens Health.

[B7] Chen SJ, Allam JP, Duan YG, Haidl G (2013). Influence of reactive oxygen species on human sperm functions and fertilizing capacity including therapeutical approaches. Arch Gynecol Obstet.

[B8] Chen H, Zhao HX, Huang XF, Chen GW, Yang ZX, Sun WJ, et al

[B9] Morrow JD, Hill KE, Burk RF, Nammour TM, Badr KF, Roberts LJ (1990). A series of prostaglandin F2-like compounds are produced in vivo in humans by a non-cyclooxygenase, free radical-catalyzed mechanism. Proc Natl Acad Sci USA.

[B10] Tremellen K (2008). Oxidative stress and male infertility – A clinical perspective. Hum Reprod Update.

[B11] Kensler TW, Wakabayashi N, Biswal S (2007). Cell survival responses to environmental stresses via the Keap1-Nrf2-ARE pathway. Annu Rev Pharmacol Toxicol.

[B12] Walczak-Jedrzejowska R, Wolski JK, Slowikowska-Hilczer J (2013). The role of oxidative stress and antioxidants in male fertility. Cent European J Urol.

[B13] Yu B, Lin H, Yang L, Chen K, Luo H, Liu J, et al (2012). Genetic variation in the Nrf2 promoter associates with defective spermatogenesis in humans. J Mol Med (Berl).

[B14] Kolahdooz M, Nasri S, Modarres SZ, Kianbakht S, Huseini HF (2014). Effects of Nigella sativa L. seed oil on abnormal semen quality in infertile men: A randomized, double-blind, placebo-controlled clinical trial Phytomedicine.

[B15] Baharin A, Hashim NE, Sonsudin F, Hashim NH (2020). Morphine and Phoenix dactylifera (dates) effects on the histological features of male rat reproductive organs. J Res Med Sci.

[B16] Saeed HSM, Osman B, El-Hadiyah TMH, Mohamed MS, Osman WJA, Abdoon IH, et al

[B17] Fallahi S, Rajaei M, Malekzadeh K, Kalantar SM (2015). Would Phoenix dactylifera pollen (palm seed) be considered as a treatment agent against Males’ infertility? A systematic review. Electron Physician.

[B18] World Health Organization (2010). WHO laboratory manual for the examination and processing of human semen.

[B19] Cooper TG, Noonan E, Von Eckardstein S, Auger J, Baker HW, Behre HM, et al (2010). World Health Organization reference values for human semen characteristics. Hum Reprod Update.

[B20] Al-Snafi AI, Bahaaldeen EF, Marbeen MI, Marbut MM (2006). The effect of date palm pollens and zinc sulphate in the treatment of human male infertility. Tikrit Journal of Pharmaceutical Sciences.

[B21] Rasekh A, Jashni HK, Rahmanian K, Jahromi AS (2015). Effect of palm pollen on sperm parameters of infertile man. Pak J Biol Sci.

[B22] Al-Dujaily SS, AL-Shahery NJ, Zabbon AA (2012). Effect of phoenix dactylifera pollen on in vitro sperm activation of infertile men. Al-Mustansiriyah J Sci.

[B23] Bahmanpour S, Panjeh Shahin MR, Talaei T, Vojdani Z, Poust Pasand A, Zareei S, et al (2006). Effect of phoenix dactylifera pollen on sperm parameters and reproductive system of adult male rats. IJMS.

[B24] Mattar AG, Adaay MH (2012). Effect of aqueous and ethanolic extracts of Tribulus terrestris, Phoenix dactylifera and Nasturtium officinale mixture on some reproductive parameters in male mice. Baghdad Sci J.

[B25] Ali-Mohamed AY, Khamis ASH (2004). Mineral ion content of the seeds of six cultivars of Bahraini date palm (Phoenix dactylifera). J Agric Food Chem.

[B26] Rahman MS, Kwon WS, Pang MG

[B27] Ebisch IMW, Thomas CMG, Peters WHM, Braat DDM, Steegers-Theunissen RPM (2007). The importance of folate, zinc and antioxidants in the pathogenesis and prevention of subfertility. Hum Reprod Update.

[B28] Nakamura BN, Lawson G, Chan JY, Banuelos J, CortÃ©s MM, Hoang YD, et al (2010). Knockout of the transcription factor NRF2 disrupts spermatogenesis in an age-dependent manner. Free Radic Biol Med.

[B29] Imai H, Hakkaku N, Iwamoto R, Suzuki J, Suzuki T, Tajima Y, et al (2009). Depletion of selenoprotein GPx4 in spermatocytes causes male infertility in mice. J Biol Chem.

[B30] Diaconu M, Tangat Y, BÃ¶hm D, KÃ¼hn H, Michelmann HW, Schreiber G, et al (2006). Failure of phospholipid hydroperoxide glutathione peroxidase expression in oligoasthenozoospermia and mutations in the PHGPx gene. Andrologia.

[B31] Schneider M, Forster H, Boersma A, Seiler A, Wehnes H, Sinowatz F, et al (2009). Mitochondrial glutathione peroxidase 4 disruption causes male infertility. FASEB J.

[B32] Peeker R, Abramsson L, Marklund SL (1997). Superoxide dismutase isoenzymes in human seminal plasma and spermatozoa. Mol Hum Reprod.

[B33] Aitken RJ, Buckingham DW, Carreras A, Irvine DS (1996). Superoxide dismutase in human sperm suspensions: Relationship with cellular composition, oxidative stress, and sperm function. Free Radic Biol Med.

[B34] Murawski M, Saczko J, Marcinkowska A, ChwiÅ‚kowska A, GryboÅ› M, BanaÅ› T

[B35] Faure C, Leveille P, Dupont C, Julia C, Chavatte-Palmer P, Alifert G, et al

[B36] Yan L, Liu J, Wu S, Zhang S, Ji G, Gu A (2014). Seminal superoxide dismutase activity and its relationship with semen quality and SOD gene polymorphism. J Assist Reprod Genet.

[B37] Ruiz-Sanz JI, Aurrekoetxea I, Matorras R, Ruiz-Larrea MB (2011). Ala16Val SOD2 polymorphism is associated with higher pregnancy rates in in vitro fertilization cycles. Fertil Steril.

[B38] Macanovic B, Vucetic M, Jankovic A, Stancic A, Buzadzic B, Garalejic E, et al

[B39] Forsberg L, LyrenÃ¤s L, Morgenstern R, de Faire U (2001). A common functional CT substitution polymorphism in the promoter region of the human catalase gene influences transcription factor binding, reporter gene transcription and is correlated to blood catalase levels. Free Radic Biol Med.

[B40] Sabouhi S, Salehi Z, Bahadori MH, Mahdavi M (2015). Human catalase gene polymorphism (CAT C-262 T) and risk of male infertility. Andrologia.

[B41] Daoud A, Malika D, Bakari S, Hfaiedh N, Mnafgui K, Kadri A, et al (2019). Assessment of polyphenol composition, antioxidant and antimicrobial properties of various extracts of date palm pollen (DPP) from two Tunisian cultivars. Arab J Chem.

[B42] Yeh CT, Yen GC (2006). Involvement of p38 MAPK and Nrf2 in phenolic acid-induced P-form phenol sulfotransferase expression in human hepatoma HepG 2 cells. Carcinogenesis.

[B43] VarÃ¬ R, D’Archivio M, Filesi C, Carotenuto S, Scazzocchio B, Santangelo C, et al (2011). Protocatechuic acid induces antioxidant/detoxifying enzyme expression through JNK-mediated Nrf2 activation in murine macrophages. J Nutr Biochem.

