# Corrigendum to “The effect of *Phoenix dactylifera* pollen on the expression of *NRF2, SOD2, CAT*, and *GPX4* genes, and sperm parameters of fertile and infertile men: A controlled clinical trial” [Int J Reprod BioMed 2021; 19: 545-558]

**DOI:** 10.18502/ijrm.v19i8.9623

**Published:** 2021-09-09

**Authors:** Soghra Fallahi, Minoo Rajaei, Mohammad Javad Hesam, Mohsen Koolivand, Kianoosh Malekzadeh

At the request of the authors the following information has been modified:

• The postal address of the correspondence was changed to “Department of Medical Genetics, Faculty of Medicine, Hormozgan University of Medical Sciences, Imam Hossein Blvd, Bandar Abbas, Iran.”
• The affiliation of the fourth author (Mohsen Koolivand) was changed to “Royan Stem Cell Technology Company, Tehran, Iran.”

The authors apologize for these mistakes.

The online version of the article was updated on September 4, 2021 and can be found at http://journals.ssu.ac.ir/ijrmnew/article-1-1922-en.html(https://doi.org/10.18502/ijrm.v19i6.9376).

